# Ultrasound shearwave elastography to characterize muscles of healthy and cerebral palsy children

**DOI:** 10.1038/s41598-021-82005-w

**Published:** 2021-02-11

**Authors:** Pauline Lallemant-Dudek, Claudio Vergari, Guillaume Dubois, Véronique Forin, Raphaël Vialle, Wafa Skalli

**Affiliations:** 1 Arts et Metiers ParisTech, Institut de Biomecanique Humaine Georges Charpak, Paris, France; 2 Physical Medicine and Pediatric Rehabilitation Department, Hôpital Armand Trousseau, APHP, Sorbonne Université, 26 avenue du Docteur Arnold Netter, 75012 Paris, France; 3 Hospital-University Department for Innovatives Therapies in Musculoskeletal Diseases DHU-MAMUTH, APHP, Sorbonne Université, Paris, France

**Keywords:** Skeletal muscle, Diagnostic markers, Movement disorders

## Abstract

Shear wave elastography (SWE) is an ultrasound technique to obtain soft tissue mechanical properties. The aim of this study was to establish the reliability of SWE in young children, define reference data on healthy ones and compare the shear modulus of healthy and spastic muscles from cerebral palsy (CP). The reproducibility is evaluated: at rest, on 7 children without any musculoskeletal pathology by 3 different operators, on 2 muscles: biceps brachii long head and medial gastrocnemius. The comparison study was made, on the same 2 muscles, at rest and under passive stretching, with a control group (29 healthy children), a spastic group (spastic muscles of 16 children from CP) and a non-spastic group (non-spastic muscles of 14 children from CP). The intra-operator reliability and inter-operator reliability, in terms of standard deviation, were 0.6 kPa (11.2% coefficient of variation (CV)) and 0.8 kPa (14.9% CV) for the biceps, respectively, and 0.4 kPa (11.5% CV) and 0.5 kPa (13.8% CV) for the gastrocnemius. At rest, no significant difference was found. Under passive stretching, the non-spastic CP biceps were significantly stiffer than the control ones (p = 0.033). Spastic gastrocnemius had a higher shear modulus than in the control muscles (p = 0.0003) or the non-spastic CP muscles (p = 0.017). CP stretched medial gastrocnemius presented an abnormally high shear moduli for 50% of patients.

## Introduction

Cerebral palsy (CP) “describes a group of permanent disorders of the development of movement and posture, causing activity limitation, that are attributed to non-progressive disturbances that occurred in the developing fetal or infant brain”^1^. One of the most frequent consequences is muscular spasticity, which must be evaluated regularly to provide suitable treatments and to measure muscular efficiency. In clinical routine, evaluation is obtained by manually stretching a specific muscle group and grading the resistance encountered, according to the movement speed, with the Modified Ashworth Scale (MAS)^2^ or the Tardieu Scale^3^. The results are reliable for two muscles groups: elbow flexors^2,3^ and ankle plantar flexors^3^. However, these grading systems do not provide a quantitative measurement of the stiffness of each muscle, which is important when treating with botulinum toxin type A injection^4^.

In vivo techniques were developed to quantify muscle stiffness noninvasively. For instance, magnetic resonance elastography gives access to the shear modulus of the muscle, a mechanical parameter indicating tissue stiffness. Basford et al*.*^5^ compared right medial gastrocnemius shear modulus between a control group (24.86 kPa) and diseased adults with spastic muscles (41.65 kPa). However, this technique is difficult to implement in terms of time and cost, and especially for young patients who are supposed to stay still for long acquisition times.

Ultrasound elastography has been used to characterize muscle stiffness. For instance, semi-quantitative sonoelastography has been used to obtain a semi-quantitative evaluation of children muscle^6–9^: at rest, spastic muscles appeared twice as stiff as healthy children muscles^8^, while a month after an intramuscular injection of botulinum toxin type A in the gastrocnemius, the stiffness of spastic muscle decreased from 3.4 to 1.5 (without unit)^9^. Acoustic Radiation Force Impulse (ARFI) has been used to evaluate muscular stiffness on children with CP^10,11^.

Shearwave elastography (SWE)^12,13^ allows quantitative estimation of tissue shear modulus with a non-invasive, non-painful and non-irradiating examination. It has been applied to evaluate muscles of CP children^14^ and compare them to healthy children^15^ on gastrocnemius. While these studies demonstrate the method’s feasibility, its reproducibility remains to be quantified. This aspect is essential for the follow-up of children, as it is necessary to distinguish the uncertainty of the technique from the evolution of the disease. Moreover, assessing reference values for typical children muscles may be useful to assess the severity of the disease.

The aim of this study was to establish the reliability of SWE in young children, define reference data on healthy ones and compare the shear modulus of healthy and spastic muscles from CP at rest and under passive stretching.

## Methods

### Participants

Before inclusion, children and parents were informed of the purpose of the study and agreed to participating and signed consent, as approved by the ethical committee (CPP 6001 Ile de France VI). Institutional Review Board approval was obtained. All the method was carried out in accordance with relevant guidelines and regulations.

Twenty-nine children younger than 15 years (12.1 ± 3.3 years old, 17 girls and 12 boys, 27 right-handed) were recruited as the healthy control group (Table 1, group 1); nine of them had mild scoliosis (Cobb angle < 20°), while fourteen had local fracture or angioma, and for these subjects the measurements were only performed on the healthy limbs. None of these subjects had any history of musculoskeletal pathology, or previous ankle, knee or elbow surgery.

**Table 1:** Characteristics of group 1 (healthy children).

N°	Age (years)	Sex	Dominant Side	Weight (kg)	Height (cm)
1	14.7	M	Right	57	168
2	15.9	M	Right	71	194
3	13.0	M	Right	42	153
4	10.8	F	Right	47	155
5	14.5	M	Right	70	165
6	12.8	M	Right	65	177
7	8.0	F	Right	24	
8	14.2	F	Right	40	159
9	13.5	F	Right	45	159
10	14.8	M	Right	50	164
11	7.3	F	Right	28	128
12	14.5	F	Right	42	149
13	15.5	F	Left	52	162
14	16.8	F	Right	45	157
15	11.6	F	Right	40	150
16	14.7	F	Left	52	172
17	13.3	F	Right	46	160
18	15.6	M	Right	65	174
19	8.5	M	Right	22	120
20	15.4	M	Right	65	176
21	12.1	F	Right	42	158
22	14.3	F	Right	43	160
23	10.5	F	Right	23.0	135
24	6.9	M	Right	25.0	126
25	10.6	M	Right	31.7	144
26	10.8	F	Right	20.7	120
27	6.8	M	Right	18.0	116
28	5.8	M	Right	17.0	112
29	6.8	M	Right	23.0	120
Mean	12.1			41.8	151.2
SD	3.3			16.3	21.4

Sixteen children affected by CP (8.3 ± 2.8 years, 6 girls and 10 boys, 6 right-handed) (Table 2) were also included. This group was constituted of: one monoparesis, four diplegia, eight hemiplegia (five of which at the right side), one child who had three limbs reached by CP and 2 children who had tetraparesia. Patients who had already received more than two intramuscular injections of botulinum toxin type A in the studied muscles were excluded, since this toxin modifies the muscle structure and biomechanical properties^16,17^. Included children had their last botulinum toxin injection at least than 4 months earlier. None of these children have had previous limb surgery. Spasticity of the studied muscles was clinically assessed with the MAS. For the upper limb, the Manual Ability Classification System for cerebral palsy (MACS)^18^ (score from 1 to 5) was calculated for each children. For the lower limb, the Gross Motor Function Classification System for cerebral palsy (GMFCS)^19^ (score from 1 to 5) was used.

**Table 2:** Characteristics of group 2 and 3 (cerebral palsy children, U = upper limb, I = lower limb, R = right, L = left).

No	Age (years)	Sex	Dominant side	Weight (kg)	Height (cm)	Spastic limbs	MACS (1 to 5)	GMFCS (1 to 5)
1	7.6	M	R			UL _ IR _ IL	3	2
2	6.9	M	L			UR _ UL _ IR _ IL	2	5
3	6.0	F	R	22	100	IR _ IL	1	2
4	5.4	M	L	17.8	111	IR	1	1
5	5.8	F	L	20		UR _ IR	2	1
6	5.6	M	L	18	107	UL _ IL	3	1
7	9.3	M	R	21		IR _ IL	1	2
8	8.8	F	R	29	133	UL _ IL	2	1
9	9.4	F	L	35	135	UR _ IR	3	1
10	7.3	F	R			IR _ IL	1	2
11	12.4	M	R	24	140	UL _ IL	2	1
12	12.7	M	_	30		UR _ UL _ IR _ IL	5	5
13	7.8	M	R	25	128	IR _ IL	1	2
14	14.8	F	L	50	153	UR_ IR	2	1
15	5.2	M	L	22	126	UR_ IR	3	1
16	8.5	M	L	33		UR_ IR	3	2
Mean	8.3			27.4	125.9			
SD	2.8			8.9	17.0			

Spastic muscles of these children were included in Group 2, while their healthy muscles were included in Group 3 (Table 2).

### Measurements

**Figure 1:**
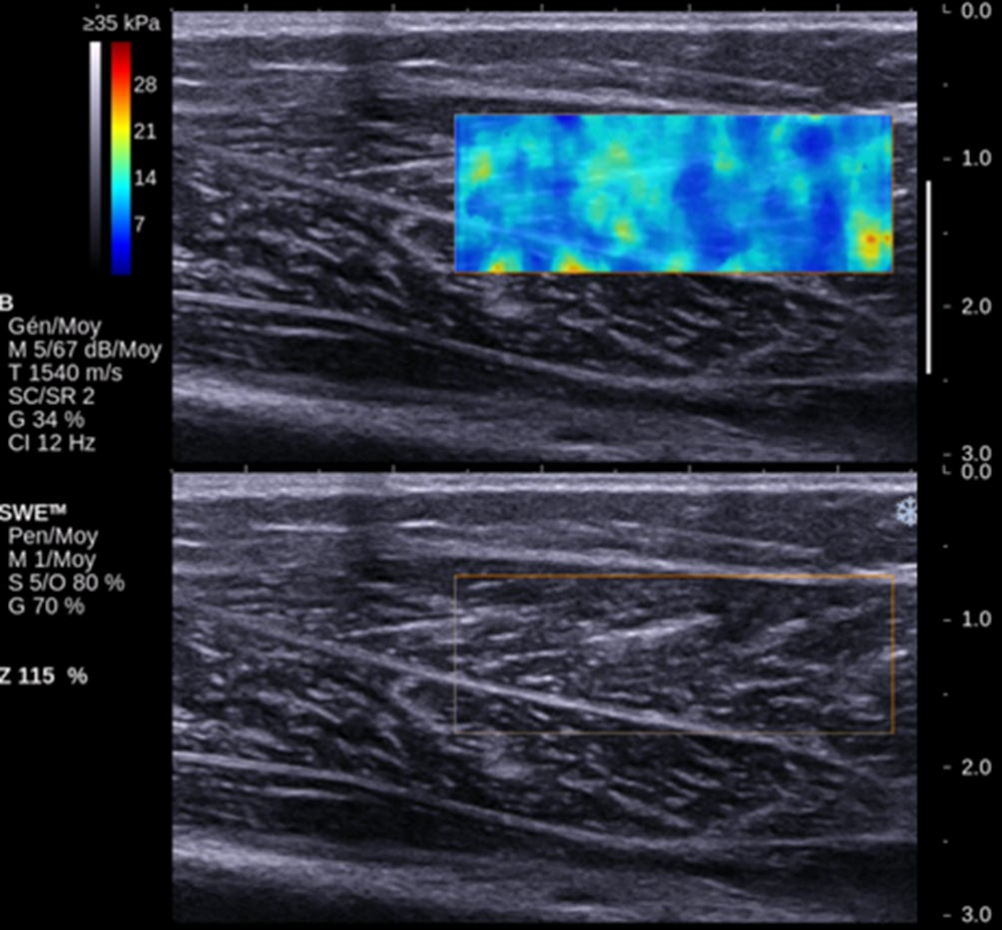
The elastography images of the medial gastrocnemius and below the ultrasonography images.

An Aixplorer ultrasound device (Supersonic Imagine, Aix-en-Provence, France), with a 50 mm 8 MHz linear probe was used to measure muscle shear modulus. The probe was applied perpendicularly to the skin and parallel to muscle fibers, and it was placed according to the anatomical landmarks described below. Four series of 8 continuous images were recorded with the device set to “general mode”, “low” spatial smoothing and 3-image temporal smoothing. Data was processed in Matlab 2016b (The MathWorks Inc., Natick, MA) as previously described^20^. Briefly, an operator defined a rectangular Region of Interest (ROI) of the elastographic image in the first frame of the series. The ROI was placed on the muscle belly, excluding skin, bone, vessels or muscle fascia (Fig. 1), but also excluding the borders of the elastographic chart to avoid border effects. Depth and size of the ROI were adapted to muscle size, in order to maximize the ROI size while avoiding the aforementioned structures. The ROI was then semi-automatically tracked in the following images; the shear modulus was space-averaged in each frame ROI, and a time-average was then calculated from all frames to obtain a single value for the series, then the four series were averaged again to obtain a single value.

### Procedure

Measurements were done on the Gastrocnemius Medialis (GCM) proximal third and the Biceps Brachii Long head (BBL) distal third for every child, both on right and left side, at Rest (_R) and under passive Stretching (_S). The subjects were asked to be as relaxed as possible.

For the measurement at rest on the GCM (GCM_R), subject was prone with a knee flexion at 90°, leg against the wall and the ankle free^21^. For the BBL (BBL_R), the subject was sitting on a chair, elbow flexion at 90°, hand in supination and the wrist fully extended. A custom elbow splint was used to maintain the position.

In order to stretch the GCM (GCM_S), the subject was prone on a table, the leg extended with the ankle hanging outside the table, while for the BBL (BBL_S), the subject was sitting on a chair and his elbow was dangling fully extended.

All measures were performed by the same operator. Four measurements (i.e., four series of 8 frames) were repeated and averaged for each muscle and each position. This protocol lasted about 15 min.

### Reproducibility

A reproducibility study was performed on seven healthy children from group 1 (5 boys and 2 girls, 8.3 ± 2.2 years old, all right-handed). All measurements were performed at rest on one upper and one lower limb; side (right or left) was randomly selected (all children were right-handed). Measurements were performed by 3 experienced operators, and each measure (i.e., each series of 8 images) was repeated 6 times. The opposite limb was examined by one operator. In other words, inter-operator reproducibility was evaluated on one upper and one lower limb, while intra-operator reliability was measured on four limbs. This protocol lasted about 40 min per subject.

### Statistical analysis

Intra and inter-operator reliability were calculated in terms of standard deviation, as recommended by the ISO 5725 standard. Intraclass Correlation Coefficient (ICC) was also calculated intra- and inter-operators for each muscle. Non-parametric Kruskal–Wallis tests and Dunn’s post-hoc analysis were used to compare the 3 groups. Dominant and non-dominant sides were compared for healthy subjects using Mann Whitney tests, while correlation between shear modulus and clinical parameters were analyzed with Spearman’s rank test. All calculations were performed in Matlab 2016b (The Mathworks, Natick, USA). Significance was set at p < 0.05.

## Results

The intra-operator repeatability was 0.6 kPa (11.2% coefficient of variation) for the BBL and 0.4 kPa (11.5%) for the GCM. The inter-operator reproducibility was 0.8 kPa (14.9%) for the BBL and 0.5 kPa (13.8%) for the GCM. ICC was equal or higher than 0.9 for each operator and both muscles, while inter-operator ICC was 0.92 for both muscles.

**Figure 2:**
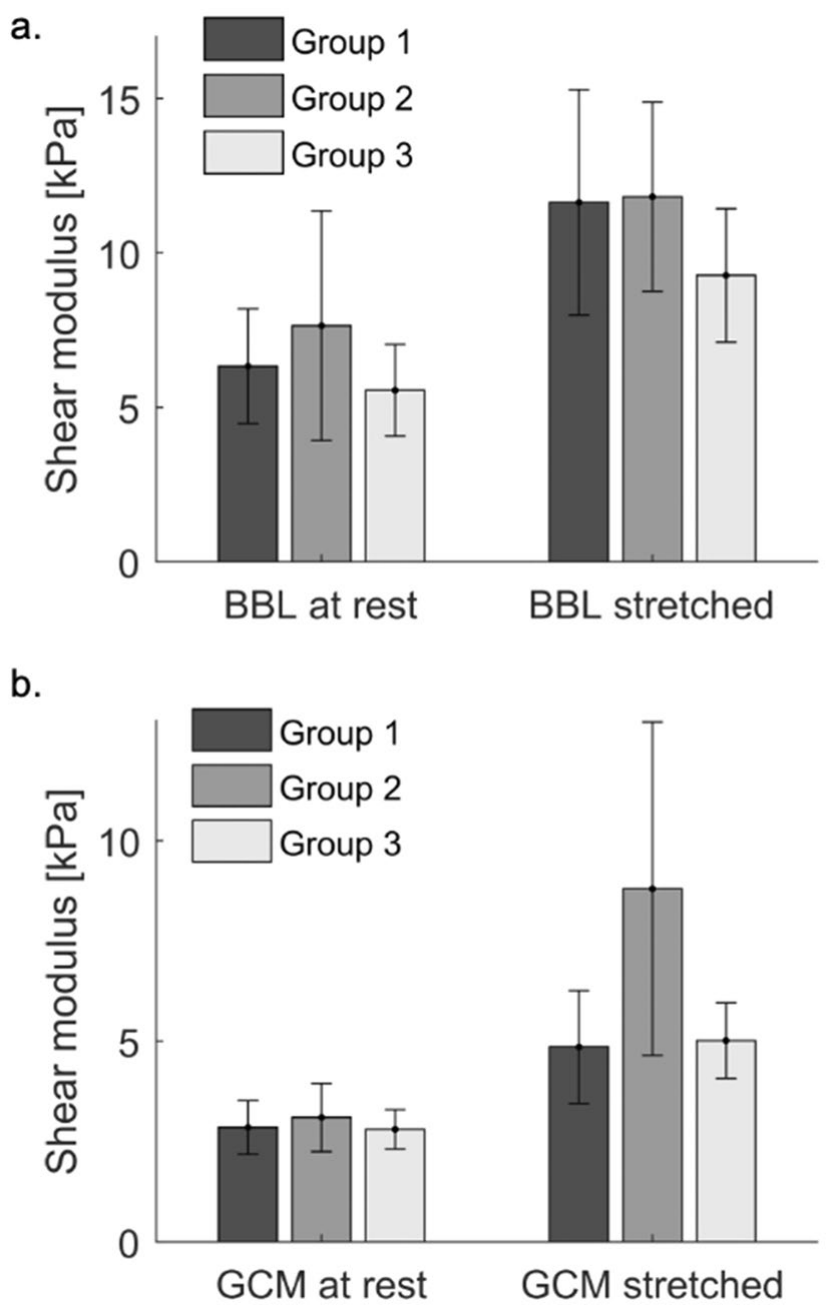
(**a**) Mean shear modulus and standard deviation per group and position for the biceps brachii long (BBL). (**b**) Mean shear modulus and standard deviation per group and position for the gastrocnemius (GCM).

Figure 2 synthesizes the mean shear moduli measured on each muscle and each position for each group. Both GCM and BBL were significantly stiffer when stretched compared to at rest in each group (p < 0.05). BBL did not show any significant difference between groups, neither at rest nor stretched (p > 0.05).

Stretched GCM was slightly stiffer in the dominant side (4.6 ± 1.4 kPa) than in the opposite side (5.1 ± 1.4 kPa, p = 0.02) for the healthy population. Stretched GCM was also significantly stiffer in group 2 (8.8 ± 4.1 kPa) than in group 1 (2.9 ± 0.7 kPa, p = 0.0001) and group 3 (5.0 ± 0.9 kPa, p = 0.05), although the latter comparison was at the limit of significance.

The ratio of stretched GCM shear modulus over modulus at rest was higher in group 2 (3.1 ± 1.8) than in groups 1 (1.8 ± 0.6, p = 0.0001) and 3 (1.9 ± 0.5, p = 0.03). In group 2, 5 patients had abnormally high shear modulus (higher than 95% subjects of group).

**Figure 3:**
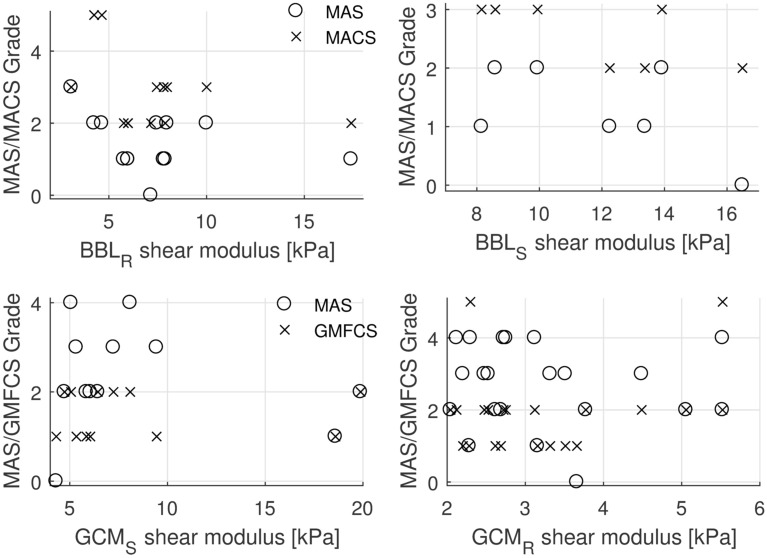
Relationship of biceps brachii long (BBL) and gastrocnemius (GCM) at rest (R subscript) or stretched (S subscript) with Modified Ashworth Scale (MAS), Manual Ability Classification System for cerebral palsy (MACS) and Gross Motor Function Classification System for Cerebral Palsy (GMFCS). No correlations were observed.

No correlations were observed between shear modulus or ratios and MAS, MACS and GMFCS scores (Fig. 3).

## Discussion

In this study, SWE reproducibility was assessed in young children, finding an uncertainty of 0.6 kPa for the BBL and 0.4 kPa for the GCM. This threshold is important when assessing possible changes with treatment or evolution disease. This sensibility could provide information about treatment efficacy or loss of efficiency before it can be detected clinically. SWE could be used as a tracking tool in addition with the clinical conclusions to precise evolutions, especially if treatment effect is poor or disability scales difficult to assess.

Spastic GCM were stiffer than healthy GCM, either if the latter is from healthy children or the contra-lateral limb of the same CP patient. Stretched GCM presented an abnormally high shear modulus for 50% of CP patients. Interestingly, GCM at rest did not show any difference between groups, nor did BBL either at rest nor stretched. This could be due to measurement reliability; indeed, inter-operator reproducibility was slightly lower in GCM than in BBL. The position adopted by patients for GCM measurements was easy to hold for disabled children. The position for the BBL was less easy to hold; the operator has to take care that the patient keeps the arm in supination. Preliminary tests showed that supination did affect measurements by slightly stretching the biceps muscle. The position used to assess the shear modulus is fundamental. The SWE permit to measure shear modulus on each muscle. The Tardieu Scale^3^ is validated on ankle plantar flexors: i.e. lateral gastrocnemius, medial gastrocnemius and soleus. To define the shear modulus muscle by muscle is an opportunity to precise spasticity local treatment (botulinum toxin injections). Furthermore, on other muscles groups which are tougher to explore clinically.

Nevertheless, results were clinically consistent, in the sense that spastic muscles were systematically stiffer than the healthy or non-spastic ones, even if sometimes with no statistical significance. Results confirm that a stretched muscle has a higher shear modulus than a muscle at rest both in healthy muscles, as previously shown in healthy^15,21–23^ and pathologic muscles^15^. Nevertheless, the latter study did not find any difference between CP children and healthy ones. The shear modulus measured in this article were smaller to those measured by Brandenburg et al*.* in lateral gastrocnemius^15^, which ranged respectively from 7.8 to 28.9 kPa versus 4.6 to 8.8 kPa in the present study. The leg and ankle position adopted in this study to stretch the GCM was similar to the “0° plantarflexion” used by Brandenburg et al*.* Consistently with their results, this position gave the largest difference between groups and variability in CP group (Fig. 2b). Also, those authors did not find any difference when values of stretched muscle were normalized on the values measured at 20° plantarflexion, which is consistent with the lack of differences found with SWS ratios measured in the present work. Dubois et al*.*^21^ reported values for GCM_R (4.7 kPa) and GCM_S (6.2 kPa) similar to those measured in this study, while Lacourpaille et al*.*^24^ reported even lower values for GCM_R (3.0 kPa).

A limitation of the present study is that the control population was older than the CP children (12.1 *versus* 8.3 years). Nevertheless, this should not have an impact on the comparison between groups since no significant age effect has been found on shear modulus, consistently with previous studies^21^. The values reported in this study on GCM and BBL shear modulus could be used as reference for children with typical development.

No correlation was found in the present cohort between the shear modulus and the disability scales, neither for BBL nor for GCM. This is consistent with the results of Brandenburg et al., who found no association between GCM shear modulus and neither GMFCS level nor MAS grade. Bilgici et al*.*, however, reported a correlation between MAS scales and shear modulus, with ARFI technique^10^. A correlation between soleus muscle MAS and shear modulus was identified by Vola et al.^25^ in an homogenous population of children affected by hemiplegic cerebral palsy. In the present study, the population was more heterogeneous as it included different limb weaknesses; this could explain the different results. Gao et al. found also a correlation between Mas and Tardieu scales in adults with post-stroke spasticity^26^ with high spasticity, in particular in cases with muscle retraction. However, a much older population was studied (59 on average, against 8.3 in this study), which is relevant since muscle elasticity changes with age. Larger studies with more subjects might be required to assess the correlation between the shear modulus and the disability scales, and to confirm the interest of measuring stretched spastic muscles to characterize the stage of neuromuscular pathologies and quantify the effects of treatment.

## Conclusion

SWE is a reliable technique to quantitatively assess muscle stiffness. This shear modulus measurement is easy to assess even on children, and it can be used in clinical routine. This study reported reference data on healthy children with typical development and new information on the biomechanical properties of spastic muscles. In particular, it appears that SWE could be more suited to assess gastrocnemius, for which large differences were observed between CP and healthy children (or healthy muscles in CP children). Measurement in biceps brachii could be less informative on their pathological status.
